# Overcoming Disembodiment: The Effect of Movement Therapy on Negative Symptoms in Schizophrenia—A Multicenter Randomized Controlled Trial

**DOI:** 10.3389/fpsyg.2016.00483

**Published:** 2016-03-31

**Authors:** Lily A. L. Martin, Sabine C. Koch, Dusan Hirjak, Thomas Fuchs

**Affiliations:** ^1^Department of Educational Science and Psychology, Free University BerlinBerlin, Germany; ^2^Department of Arts Therapies and Therapy Science, Alanus University AlfterAlfter, Germany; ^3^Department of Dance and Movement Therapy, School of Therapeutic Sciences, SRH University HeidelbergHeidelberg, Germany; ^4^Department of General Psychiatry, Centre for Psychosocial Medicine, Academic Medical Center, University of HeidelbergHeidelberg, Germany

**Keywords:** schizophrenia, negative symptoms, embodiment/disembodiment, Dance Movement Therapy (DMT), Body Psychotherapy (BPT), randomized controlled trial, multiple imputation

## Abstract

**Objective:** Negative symptoms of patients with Schizophrenia are resistant to medical treatment or conventional group therapy. Understanding schizophrenia as a form of *disembodiment* of the self, a number of scientists have argued that the approach of *embodiment* and associated embodied therapies, such as Dance and Movement Therapy (DMT) or Body Psychotherapy (BPT), may be more suitable to explain the psychopathology underlying the mental illness and to address its symptoms. Hence the present randomized controlled trial (DRKS00009828, http://apps.who.int/trialsearch/) aimed to examine the effectiveness of manualized movement therapy (BPT/DMT) on the negative symptoms of patients with schizophrenia.

**Method:**A total of 68 out-patients with a diagnosis of a schizophrenia spectrum disorder were randomly allocated to either the treatment (*n* = 44, 20 sessions of BPT/DMT) or the control condition [*n* = 24, treatment as usual (TAU)]. Changes in negative symptom scores on the Scale for the Assessment of Negative Symptoms (SANS) were analyzed using Analysis of Covariance (ANCOVA) with Simpson-Angus Scale (SAS) scores as covariates in order to control for side effects of antipsychotic medication.

**Results:**After 20 sessions of treatment (BPT/DMT or TAU), patients receiving movement therapy had significantly lower negative symptom scores (SANS total score, blunted affect, attention). Effect sizes were moderate and mean symptom reduction in the treatment group was 20.65%.

**Conclusion:**The study demonstrates that embodied therapies, such as BPT/DMT, are highly effective in the treatment of patients with schizophrenia. Results strongly suggest that BPT/DMT should be embedded in the daily clinical routine.

## Introduction

Despite more than a century of research and major improvements regarding anti-psychotic medication as well as psychological treatment, the majority of the burden of schizophrenia is un-avertable in the light of current knowledge. Three quarters of patients suffering from schizophrenia experience recurrent and persistent symptoms with a substantial impact on their daily and social life and full remissions are rare (Deutsche Gesellschaft für Psychiatrie Psychotherapie und Nervenheilkunde (DGPPN), 2006; National Collaborating Centre for Mental Health (NCCMH), 2014). In particular persistent negative symptoms are an unmet therapeutic need (Kirkpatrick et al., 2006). They frequently are associated with treatment-refractory processes, illness chronicity and therefore particularly poor function and quality of life (Kirkpatrick et al., 2006; Blanchard et al., 2011). Pharmacological interventions, which have been the mainstay of treatment since their introduction in the 1950s, have a number of limitations, such as high incidence of disabling side effects and poor adherence to treatment (NCCMH, 2014). Moreover, no drug has yet received Food and Drug Administration (FDA) approval for an indication of negative symptoms (Kirkpatrick et al., 2006).

Andrews et al. (2003) conclude that even by means of optimal available treatment only 22% of estimated disability-adjusted life-years (DALYs) of schizophrenic patients can be regained. This may be due to the disorder's substantial clinical, pathological, and etiological heterogeneity and a consequent academic dissent on its origin and symptom structure. In particular the concept of “negative symptoms” is the cause of a long-standing debate in regard to its differentiation to and interrelation with positive symptoms (Carpenter et al., 1988; Kukla and Gold, 1991; Arango et al., 2004; Blanchard and Cohen, 2006). Additionally, the list of symptoms generally regarded as “negative” is extensive and controversial (Andreasen and Olsen, 1982; Carpenter et al., 1985; Kukla and Gold, 1991).

The lack of conventional treatment options, however, has paved the way for acceptance of a more broadly-based, interdisciplinary and innovative approach: Combining theoretical ideas and practical implications from the areas of philosophy, psychology, psychiatry, and neuroscience, the approach of *embodiment* denominates a field of research, which investigates the circular interaction of mind, brain, organism and environment in the etiology of psychiatric disorders. Underpinned by phenomenological concepts and neuroscientific findings, the *embodiment paradigm* focuses on the implicit functioning of the body in everyday perception and performance (Merleau-Ponty, 1962; Gallagher, 2005; Fuchs and Schlimme, 2009; Koch and Fuchs, 2011; Fuchs, 2012; Fuchs and Koch, 2014). The “lived” or “subject body” is understood as a transparent medium or background to our experience of the world (“mediated immediacy”); (Fuchs, 2005). By constituting our prereflective sense of self and agency, it allows us to attune to the environment and in particular to others through a shared intercorporeality (Fuchs and Schlimme, 2009).

Against this background, schizophrenia is regarded as a fundamental disturbance of the embodied self, or a *disembodiment*. This includes a weakening of the basic sense of self, a disruption of implicit bodily functioning and, as a result, a disconnection from the inter-corporeality with others (Parnas, 2003; Sass and Parnas, 2003; Fuchs, 2005; see Box 1 for an explication of phenomenological terms and their usage in the explanation of schizophrenic symptoms). In contrast to current neuropsychological theories, which attribute the core disturbance of the illness to higher order cognitive processes (“theory of mind,” “meta-representation”; Frith, 2004), the phenomenological approach locates the main disorder on a lower level, emphasizing basic abnormalities of bodily mediated consciousness that underlie and antecede the disparate assortment of symptoms in schizophrenia. It thereby not only allows for positive and negative symptoms and their interrelation to be seen from an integrative angle (Sass and Parnas, 2003; Fuchs and Schlimme, 2009) but more generally for dualistic concepts of mind and body to be overcome.

Box 1 **Phenomenological terms in the explication of symptoms in schizophrenia.**

**Table:** 

**The body as transparent medium**
In our perceiving of and interacting with the world, the body functions as an implicit medium, which itself remains in the background of awareness. We perceive and act “through” the body without taking notice of it (mediated immediacy).
**Pre-reflective/basic sense of self**
The immediate self-givenness or “mineness” of all experience as being embedded in a background feeling of the body, before any reflective relation to oneself; the certainty that my experiences are my own.
**Pre-reflective/basic sense of agency**
The experience of being a source of spontaneity and being the one who initiates an action (“mineness” of acting).
**Intercorporeality**
The pre-reflective and non-symbolic interaction with others in face-to-face encounters, based on the implicit mutual intertwinement of expressions, gestures, and postures, mediating a basic emotional understanding of others.
**Somatic sensations as tacit medium of affectivity**
In every emotion interoceptive and proprioceptive sensations function as a “bodily resonance,” which lends the emotion its self-affecting or “felt” character, though usually remaining in the background. Only in intense emotions, the bodily sensations become conscious.
**Disembodiment**
The disturbance and alienation of the habitual or implicit bodily functioning on the level of perception, action or intercorporeality.
**Fragmentation of thought and action**
Disintegration of the synthetic units of intentional thinking and goal-directed action, leading to the appearance of single fragments of thought or movement in the experiential field.
**Hyperreflexivity**
Hyper-awareness of normally implicit or background functions of the body, often combined with an exaggerated reflection on oneself and the meaning of one's experiences.
Phenomenological and embodiment based approaches regard schizophrenia as a fundamental disturbance of the embodied self, or a *disembodiment*. A disembodied self does not “inhabit” the body any more, in the sense of using for granted its implicit structure, emotional resonance and automatic performances (Fuchs, 2005, 2012). The loss of tacit self-awareness results in an alienation of somatosensory perception, emotional expression, movement, and action: Somatic sensations usually experienced as the tacit medium of an attitude or affect are detached from their motivational context (Sass, 2000), leaving the patient incapable of making sense of felt emotions as well as adequately expressing or following them. Negative symptoms such as flat affect and loss of drive and desires may be regarded as a result of this disembodied affectivity. Moreover, units of meaningful actions such as reading or getting dressed are fragmented, resulting in a pathological explication and a hyper-reflexive awareness to normally tacit aspects of everyday life (Sass and Parnas, 2003; Fuchs, 2005). Eventually, in an advanced state of the illness the subject might lose the sense of agency for his or her own emotions or actions, leading to delusions of manipulation or alien control (positive symptoms; Sass and Parnas, 2003; Fuchs and Schlimme, 2009; Fuchs, 2015).

By conceptualizing body, mind, action, and perception as a unity, embodiment researchers stress the need to target body experiences in order to change emotions and behavior, specifically in the case of severe mental disorders when verbal dialog can be difficult (Röhricht, 2009; Koch and Fischman, 2011; Koch and Fuchs, 2011; Fuchs and Koch, 2014; Tschacher et al., 2014). According to the approach, affect and cognition are not only reflected in body posture and movement but also considerably influenced by them (Koch and Fuchs, 2011). For example, the mere taking on of a dominant vs. a submissive body posture has been shown to cause changes in self-experience, testosterone levels, saliva secretion, and risk-taking behavior (Carney et al., 2010). Approach movements such as arm flexion, caused subjects to rate arbitrary Chinese ideographs more positively than when doing avoidance movements, such as arm extension (Cacioppo et al., 1993). Similarly, different movement qualities (sharp vs. smooth movements) and rhythms in handshakes have been shown to influence affective, cognitive and social perception, and reaction toward an unknown examiner (Koch, 2011).

Embodied therapies such as Body Psychotherapy (BPT) or Dance and Movement Therapy (DMT) apply embodiment concepts in the service of the client. While BPT has developed from a psychodynamic background to an independent therapeutic approach, DMT understands itself as one of the creative arts therapies (dance, drama, music, art, and poetry therapy). Working mostly in a one-on-one context, BPT combines specific body-oriented, non-verbal interventions with insight-oriented, verbal techniques to obtain behavior modification (Röhricht, 2009). DMT beyond that focuses on the therapeutic use of movement to further emotional, cognitive, physical, and social integration of the individual (American Dance Therapy Association, 2015). It primarily takes place in group settings. By emphasizing the meaning of sensorimotor experience and body motion for cognition, affect, and (inter)action both disciplines operate at the center of emotional processing and self-regulation and use mutual mechanisms of therapeutic change. Manualized movement therapy for treating negative symptoms in chronic schizophrenia (a combination of BPT and DMT and therefore named BPT/DMT throughout this paper) applies body-oriented interventions to reconstruct a basic and coherent ego-structure and to strengthen self-referential processes (Röhricht and Papadopoulos, 2010). By integrating sensory awareness and movement techniques, it targets core body image disturbances (boundary loss, disembodiment) and widens the range of responsive, expressive and communicative behaviors (movement and speech) in order to reduce emotional withdrawal (Röhricht et al., 2011).

According to the guidelines of the National Institute for Clinical Excellence (NICE), “arts therapies,” such as DMT, BPT, art psychotherapy, drama, and music therapy are currently the only interventions (both psychological and pharmacological) to demonstrate consistent efficacy in the reduction of negative symptoms [NCCMH, 2014]. However, the existing empirical evidence is still weak. A Cochrane Review by Xia and Grant on movement therapy for schizophrenia found only one randomized controlled trial, which met the rigorous quality standards of the review groups (Xia and Grant, 2009). The study, conducted by Röhricht and Priebe (2006), investigated the effects of BPT in schizophrenia and found a significant reduction of negative symptoms in patients receiving BPT compared to patients receiving supportive counseling (Röhricht and Priebe, 2006). Effect sizes were large and consistent even at a 4 month follow up. An open uncontrolled trial, conducted by the same authors, led to similar results (Röhricht et al., 2011). A major limitation of the available evidence is that it originates from a small exploratory trial with one BPT therapist, based only at one institution. It remains unclear whether the effect can be replicated across different therapists, settings and samples (Röhricht and Priebe, 2006). Both, Xia and Grant (2009) as well as NCCMH (2014) urge further adequate research. In order to increase high quality evidence for the efficacy of embodied movement therapy in the treatment of schizophrenia, our randomized controlled trial aimed to replicate and expand upon Röhricht and Priebe's findings by using an increased sample size, several therapists and patients coming from different medical centers.

In this sense, we hypothesized that:

- Manualized movement therapy (BPT/DMT) generally reduces negative symptoms of schizophrenia, when controlled for extrapyramidal side effects of antipsychotic medication.
- Manualized movement therapy (BPT/DMT) particularly reduces non-cognitive core negative symptoms that are associated with a disembodied awareness of the self, such as loss of emotional resonance or blunted affect (Liddle, 2000).

## Methods

### Study design

The trial constituted the randomized controlled part of a larger study, conducted at the Department of General Psychiatry in Heidelberg, Germany, as part of the EU-project “Toward an Embodied Science of Intersubjectivity (TESIS).” See the Supplementary Material for the project flyer. It was approved by the local ethics committee of the Medical Faculty of the University of Heidelberg, registered with DRKS (German Clinical Trials Register: DRKS00009828, http://apps.who.int/trialsearch/) and funded by the TESIS project as well as private sponsors. Data was assessed and analyzed following a double-blind, two factorial design, comprising the factors *Time* (before and after the treatment) and *Group* (treatment or control group).

### Recruitment procedure and randomization

Participants were consecutively recruited between 2012 and 2014 from three medical centers: The Centre for Psychosocial Medicine in Heidelberg, the Psychiatrisches Zentrum Nordbaden (PZN) in Wiesloch and the Johannes-Diakonie in Mosbach. Selection criteria were: (1) age between 14 and 65 years; (2) diagnosis of a schizophrenia spectrum disorder (IDC-10: F20.x, F25.x); (3) outpatient; (4) stable medication. Patients were excluded from the study (not necessarily from the therapeutic intervention), if (1) they were in a phase of acute psychosis, (2) they had a history of brain trauma, neurological, or internistic disease, affecting their movement abilities, (3) they had shown alcohol or substance abuse or dependency within 24 months prior to participation or were diagnosed with a substance induced psychosis, (4) if they had an IQ < 70, and (5) if there were pronounced language barriers.

Initial diagnoses stemmed from psychiatrists or psychotherapists, who were not involved in the study. Screening, baseline, and outcome assessment was conducted by clinical raters trained in the use of the assessment instruments: medical doctors and psychologists. Inclusion diagnoses were confirmed via the German version of the Structured Clinical Interview for DSM-IV (Wittchen et al., 1997) and reviews of hospital case notes. All eligible patients were randomly allocated to either the treatment or the control group and invited for an extensive diagnostic interview prior to and after the intervention. Clinical raters were blind to the hypothesis of the study and to the group allocation. Randomization was done by one of the project coordinators, who was not involved in data assessment and analysis. Computer based block randomization (Suresh, 2011) via Excel was used in order to form small treatment groups of up to eight patients. Allocation ratio of treatment and control group was intended to be 2:1. To ensure blindness of the raters participants were informed of their allocated group via a sealed envelope after baseline assessment. All participants gave their written informed consent prior to the start of the study. In this study no participants under the age of 18 were included. Furthermore, participants were informed of the possibility to withdraw consent without any obligation to declare specific reasons. After the completion of the outcome assessment, participants received an expense allowance of 20 Euros.

### Treatment conditions

While the treatment group received 10 weeks (twenty sessions) of additional movement therapy (BPT/DMT) the control group waited during this time, receiving treatment as usual (TAU) by the respective outpatient department. Patients initially allocated to the control group had the opportunity to attend BPT/DMT after the assessment period. About 60% made use of this offer. TAU comprised medical treatment only. All patients received treatment with a single antipsychotic agent according to their psychiatrist's choice. Antipsychotic treatment remained stable during 10 weeks of BPT/DMT and included second generation antipsychotics such as clozapine, olanzapine, aripiprazole, or risperidone. Movement therapy was conducted following the manual of Röhricht and Papadopulos “Body oriented psychological therapy for chronic schizophrenia” (Röhricht and Papadopoulos, 2010). The manual, specifically designed for patients suffering from schizophrenia, aims to increase body awareness, decrease dysfunctional self-perception thereby promoting affect expression and interpersonal responsiveness. It consists of different individual, pair or group exercises, which are structured in five parts that are regularly repeated in each session: (1) Opening circle, (2) Warm-up, (3) Structured task, (4) Creative movement, (5) Closing circle (for a detailed description of the parts see Röhricht and Papadopoulos, 2010 and Röhricht and Priebe, 2006). Treatment sessions of 90 min took place twice a week in groups of up to eight persons. They were conducted by accredited dance movement therapists (DMTs) trained in the usage of the manual in a 3-days workshop with Röhricht and Papadopulous. The DMTs were otherwise not involved in the patients' care. Each dance movement therapist was supported by a co-therapist (students of either DMT or psychology), also specifically trained to assist within the sessions.

### Clinical assessment

Demographics as well as the psychiatric and medical history of the clinical sample were retrieved from medical records. Extensive clinical assessment of all participants (treatment and control group) took place prior to and after the 10-week intervention period in the respective psychiatric institution. Three to six month follow-up assessment was planned, but not realized due to high drop-out rates.

As part of the larger TESIS study a number of psychopathological symptoms as well as the general functioning of the patients were assessed: the amount of positive symptoms was recorded using the Scale for the Assessment of Positive Symptoms (SAPS; Andreasen, 1984b). The overall severity of psychopathological symptoms was assessed using the Brief Psychiatric Rating Scale (BPRS; Overall and Gorham, 1962). Finally the social, occupational and psychological functioning of participants was assessed using the Global Assessment of Functioning (GAF) Scale (Hall, 1995).

The primary outcome measure of the sub-study at hand was the amount of negative symptoms of the participants. It was assessed using the Scale for the Assessment of Negative Symptoms (SANS; Andreasen, 1984a). The observation scale captures the global level of negative symptoms (hypothesis a) by taking into account five subsets of symptoms: (1) blunted affect, (2) alogia, (3) abulia/avolition, (4) anhedonia and (5) diminished attention. Each subset consists of several items (in total 24), which are rated from absent (a score of 0) to severe (a score of 5). Because it focuses on non-cognitive negative symptoms, which are associated with a loss of embodied self-awareness, we paid specific attention to subscale (1) blunted affect (hypothesis b). Sufficient internal consistency (Cronbach's α = 0.89) and external validity of the scale have been previously reported (Andreasen, 1982; Peralta et al., 1995; Rabany et al., 2011).

Because negative symptoms can be evoked or enhanced by antipsychotic medication, we recorded extrapyramidal side effects as a control variable, using the Simpson-Angus Scale (SAS/EPS in German; Simpson and Angus, 1970). The scale is composed of ten items, which are rated from normal (a score of 0) to extreme (a score of 4), and provides a global evaluation of the extrapyramidal syndrome. The assessment scales of the primary outcome measures can be seen in the Supplementary Material.

### Data analysis

All data were analyzed using the Statistical Package for the Social Sciences (SPSS version 22.0 for OSX 10.8, SPSS Inc., Chicago, IL, USA).

#### Sample size

Power calculations revealed that a number of 90 participants was required to detect moderate to large treatment effects of 0.3–0.5 (Cohen, 1988) with a two-tailed α of 0.5 and a power of 0.8. Because structural and institutional limitations and illness-induced motivational restraints in the schizophrenic population were expected to lead to a considerable number of drop-outs, a power of 0.8 was considered sufficient and recruitment aimed to reach the upper edge of 90 participants in total.

#### Missing data

**Figure 1:**
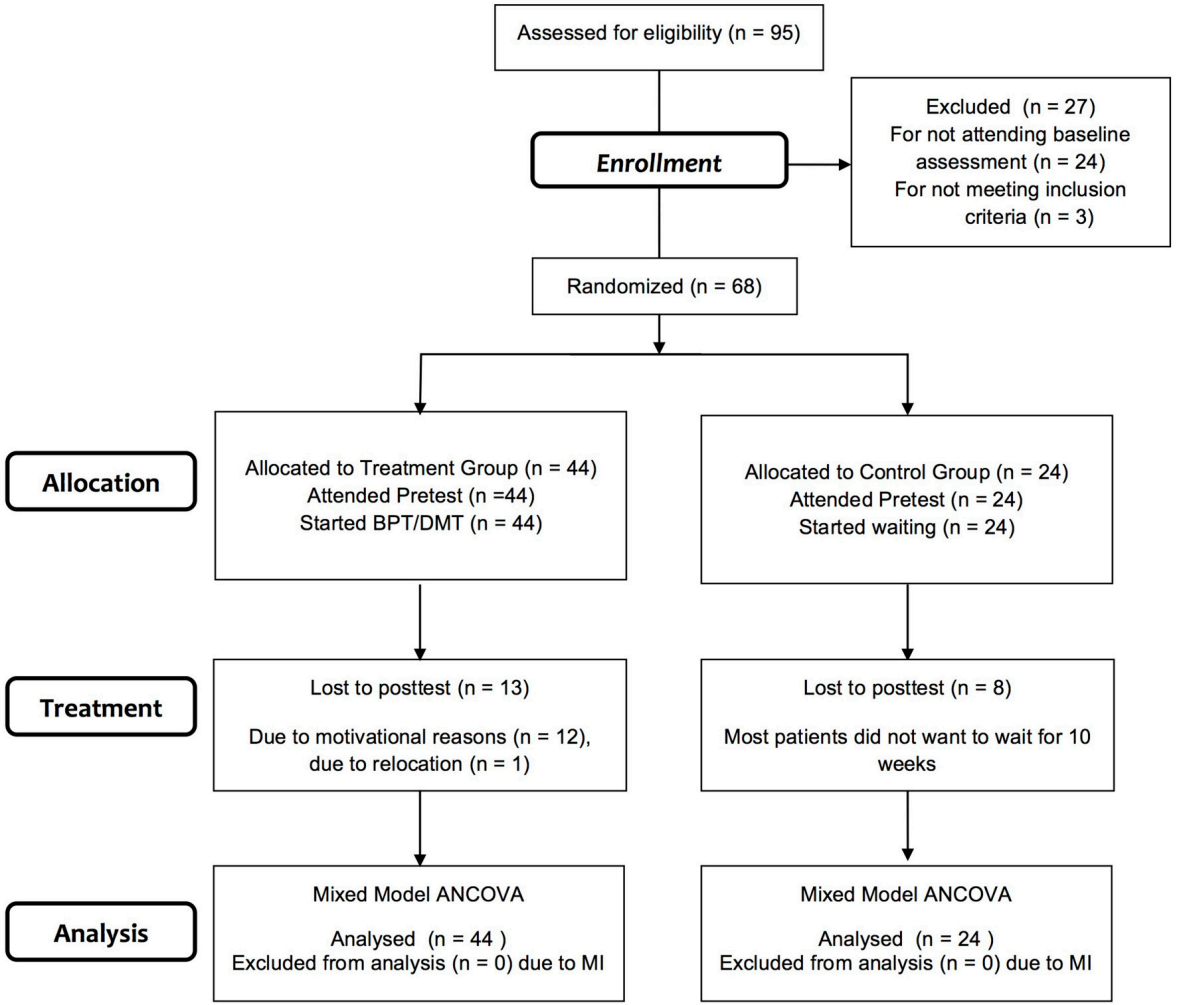
**Participant flow chart following consolidated standards of reporting trials guidelines**. BPT/DMT, manualized movement therapy following Röhricht and Papadopoulos (2010); ANCOVA, Analysis of Covariance; MI, Multiple Imputation.

As expected a large amount of missing data (up to 43.2%, see Table 1) needed to be accounted for. Most data was missing due to drop-outs of participants (drop-out rate: 30.9%, see Figure 1). In order to prevent the exclusion of a large number of the original sample and a substantial loss of precision and power, Multiple Imputation (MI) was performed. MI allows for uncertainty due to missing data by using a regression model to estimate the missing data multiple times. It thereby creates several different plausible imputed data sets. The statistical analysis of interest is performed on each completed data set separately and separate results are appropriately combined (pooling; Sterne et al., 2009; Hayati Rezvan et al., 2015). MI can only be done, if data is missing completely at random (MCAR) or missing at random (MAR), that is if there is no systematic difference between the missing values and the observed values or if any systematic difference can be explained by one or more of the assessed variables (Sterne et al., 2009; Enders, 2010; Eid et al., 2013). MCAR was tested by performing Little's Test for treatment and control group respectively (treatment group: χ^2^ = 66.76, *df* = 69, *p* = 0.55; control group: χ^2^ = 44.08, *df* = 51, *p* = 0.74). MAR was tested by calculating logistic regressions of assessed demographic variables, such as group (treatment/control group), age, nationality or family status, on specifically created “missing-variables.” Since none of the variables proved significant MI was performed comprising all assessed demographic and clinical variables as predictor variables and primary outcome measures as well as further outcome measures of the larger study as criterion variables (see Table 2). Because previous pilot trials found no significant relation between changes of positive and negative symptoms in the respective sample (Heidbüchel, 2013) we omitted values of the SAPS to increase specificity and reduce bias of the imputation model. Due to practical and computational capacities, imputation was performed on scale rather than on component level, this is demonstrated in Table 2. Twenty imputed data sets were created using the fully conditional specification imputation method with 10 iterations and 1000 parameters allowed in the imputation model.

**Table 1:** **Rates of missing data for each variable imputed**.

	**Treatment**^**a**^		**Control**^**b**^	
	**Missing**	**%**	**Missing**	**%**
**BPRS-TS**				
T1	5	11.4	6	25
T2	13	29.5	6	25
**BPRS-SNS**				
T1	5	11.4	6	25
T2	13	29.5	6	25
**GAF**				
T1	7	15.9	10	41.7
T2	17	38.6	11	45.8
**SANS-TS**				
T1	1	2.3	3	12.5
T2	14	31.8	5	20.8
**(1) SANS-BA**				
T1	1	2.3	3	12.5
T2	12	27.3	5	20.8
**(2) SANS-ALOGIA**				
T1	1	2.3	3	12.5
T2	12	27.3	5	20.8
**(3) SANS-ABULIA**				
T1	1	2.3	3	12.5
T2	12	27.3	5	20.8
**(4) SANS-ANHEDONIA**				
T1	1	2.3	3	12.5
T2	12	27.3	5	20.8
**(5) SANS-ATTENTION**				
T1	1	2.3	3	12.5
T2	14	31.8	5	20.8
**SAS-TS**				
T1	3	6.8	7	29.2
T2	19	43.2	8	33.3

BPRS-TS, Brief Psychiatric Rating Scale Total Score; BPRS-SNS, Brief Psychiatric Rating Scale Subscale Negative Symptoms (BPRS-SNS comprises Item 3, 13, and 16); GAF, Global Assessment of Functioning; SANS-TS, Scale for the Assessment of Negative Symptoms Total Score; SANS-BA, Scale for the Assessment of Negative Symptoms Subscale Blunted Affect, SAS-TS, Simpson Angus Scale Total Score; T1, Measurement Time 1 prior to the treatment; T2, Measurement Time 2 after the treatment.

a n = 44.

b n = 24.

**Table 2:** **Variables used in the imputation model**.

**Predictor variables**	**Criterion variables**
Group	BPRS-TS
Testing place	BPRS-SNS
Sex	GAF
Age	SANS-TS
Nationality	SANS-BA
Relationship status	SANS-Alogia
Family status	SANS-Abulia
Education	SANS-Anhedonia
Mother tongue	SANS-Attention
BPRS-TS	SAS-TS
BPRS-SNS	
GAF	
SANS-TS	
SANS-BA	
SANS-Alogia	
SANS-Abulia	
SANS-Anhedonia	
SANS-Attention	
SANS global rating BA	
SANS global rating alogia	
SANS global rating abulia	
SANS global rating anhedonia	
SANS global rating attention	
SANS-SS	
SAS-TS	

All clinical variables were assessed twice: prior to and after the treatment. Therefore, scores of clinical variables were included for both measurement times (T1 and T2) respectively; BPRS, Brief Psychiatric Rating Scale; GAF, Global Assessment of Functioning; SANS, Scale for the Assessment of Negative Symptoms; SAS, Simpson-Angus Scale; TS, Total Score; SNS, Subscale Negative Symptoms (BPRS-SNS comprises item 3, 13, and 16); BA, Blunted Affect; SS, Summary Score (SANS-SS is the sum of all global rating items).

#### Main analysis

Differences in the change of negative symptom scores between treatment and control group were tested using Mixed Model Analysis of Covariance (ANCOVA) with *Time* as “within subject factor” and *Group* as “between subject factor” and with SAS composite scores as covariates in order to control for side effects of medication. ANCOVAs were undertaken for the overall SANS score (SANS-TS) and for each subscore respectively [(1) SANS-BA, (2) SANS-Alogia, (3) SANS-Abulia, (4) SANS-Anhedonia, (5) SANS-Attention]. Therefore, α values were corrected for the number of tested scores (*N*) using the Bonferroni method: α = 0.05/*N*. The corrected threshold was set to 0.01.

Because SPSS does not provide an automatized way to pool results of a Mixed Model ANCOVA done on multiply imputed data, a macroinstruction (macro) of Van Ginkel (van Ginkel, 2014) was used [for a detailed account on this method see also van Ginkel and Kroonenberg, 2014].

Contrasts comparing the two groups at both times of measurement and each group over time were computed using *t*-tests. To take into account existing baseline differences in the primary outcome variables, we performed an outlier analysis, logistic regressions as well as subsequent ANCOVAS, with respective SANS baseline scores as covariates.

## Results

### Sample

A total of 95 patients were referred for inclusion, 68 of whom met the inclusion criteria, consented to participation and were randomized to the two groups (see Figure 1). During the treatment period, there was a large number of drop-outs: in total 21 patients (30.9%). Most patients withdrew from the study due to a lack of motivation and one patient moved to another city. Demographic and clinical characteristics of the study sample (*n* = 68) are shown in Table 3. The sample consisted of 32 women and 36 men in total, with a mean age of 39.84 years (*SD* = 10.35) and with a mean duration of 15.92 (*SD* = 10.00) years of schizophrenic symptoms. Most participants were German, spoke German as their first language, lived without a partner and children and had at least completed secondary school and some kind of vocational training (see Table 3 for exact numbers). Treatment and control group did not differ significantly in any of the demographic variables.

**Table 3:** **Demographic and clinical data of the study participants**.

		**Treatment^a^**	**Control^b^**
Gender	F/M	19/25	13/11
Age	$\bar{x}$ (years)/*SD*	41.05/10.84	37.52/9.14
Duration of illness	$\bar{x}$ (years)/*SD*	16.13/11.34	15.56/7.88
Treatment Place	Heidelberg	23 (52.27%)	19 (79.16%)
	Wiesloch	16 (36.36%)	5 (20.83%)
	Mosbach	5 (11.36%)	0
Medication	Atypical	44	24
	Typical	0	0
Nationality	German	34 (72.27%)	18 (75%)
	Other	2 (4.54%)	2 (8.33%)
	Two nationalities	1 (2.27%)	2
	Missing	7 (15.91%)	2
Mother Tongue	German	33 (75%)	17 (70.83%)
	English	1 (2.27%)	1 (4.17%)
	Spanish	1	2 (8.33%)
	Other	2 (4.54%)	2
	Missing	7 (15.9%)	2
Education	No degree	3 (6.81%)	2
	Lower secondary school	2 (4.54%)	4 (16.67%)
	Middle school	8 (18.18%)	1 (4.17%)
	High school	4 (9.1%)	3 (12.5%)
	Apprenticeship	13 (29.54%)	7 (29.17%)
	Studies	7 (15.9%)	5 (20.83%)
	Missing	7	2 (8.33%)
Relationship Status	No partner	20 (45.45%)	11 (45.83%)
	In relationship	14 (31.18%)	9 (37.5%)
	Missing	10 (22.72%)	4 (16.67%)
Children	Children	7 (15.9%)	2 (8.33%)
	No children	21 (47.72%)	16 (66.67%)
	Missing	16 (36.36%)	6 (25%)

The duration of the illness was computed in 2015.

a n = 44.

b n = 24.

### Changes in negative symptoms

Psychometric properties of the primary outcome variables are summarized in Table 4. Because a large fraction of missing data was imputed, psychometrics are given for (a) observed and (b) imputed values, respectively. Means and standard deviations of (a) and (b) did not display any notable differences. Imputed values therefore could be regarded as reasonable and inferences from pooled analysis as valid. So far there are no clinically meaningful cut-off values for the SANS. According to Levine and Leucht (2013) participants' overall severity of negative symptoms were mild to moderate: up to 70 on a scale from 0 to 120.

Table 4**Psychometric properties of the major study variables (A) before MI and (B) after MI**.

**Table:** 

	**Treatment**				**Control**			
**(A)**	***n***	***M***	***SD***	**SEM**	***n***	***M***	***SD***	**SEM**
**SANS-TS**								
T1	43	28.72	16.06	2.45	21	17.48	12.92	2.82
T2	30	22.37	12.11	2.21	19	25.16	14.43	3.31
**(1) SANS-BA**								
T1	43	9.44	6.14	0.94	21	5.43	7.26	1.58
T2	32	7.19	5.27	0.93	19	8.26	5.14	1.18
**(2) SANS-ALOGIA**								
T1	43	3.00	3.29	0.50	21	1.43	1.86	0.41
T2	32	2.25	2.63	0.46	19	2.21	3.24	0.74
**(3) SANS-ABULIA**								
T1	43	5.05	3.50	0.53	21	4.43	3.17	0.69
T2	32	3.84	2.41	0.43	19	5.16	4.32	0.99
**(4) SANS-ANHEDONIA**								
T1	43	6.88	5.31	0.81	21	3.81	3.23	0.71
T2	32	5.88	3.82	0.68	19	5.84	4.54	1.04
**(5) SANS-ATTENTION**								
T1	43	4.35	2.84	0.43	21	2.38	2.13	0.47
T2	30	2.63	2.43	0.44	19	3.68	3.30	0.76
**SAS-TS**								
T1	41	4.85	3.79	0.59	17	2.24	2.33	0.57
T2	25	2.88	2.24	0.45	16	3.19	2.51	0.63

**Table:** 

	**Treatment**^a^			**Control**^b^		
**(B)**	**M**	**SD**	**SEM**	**M**	**SD**	**SEM**
**SANS-TS**								
T1	28.62	15.91	2.40	18.44	12.49	2.55
T2	22.71	10.37	1.56	24.86	13.03	2.66
**(1) SANS-BA**								
T1	9.40	6.10	0.92	5.76	7.01	1.43
T2	7.33	4.84	0.73	8.12	4.81	0.98
**(2) SANS-ALOGIA**								
T1	2.97	3.25	0.49	1.61	2.01	0.41
T2	2.32	2.52	0.38	2.21	2.99	0.61
**(3) SANS-ABULIA**								
T1	5.05	3.45	0.52	4.44	3.04	0.62
T2	4.00	2.52	0.38	5.01	4.02	0.82
**(4) SANS-ANHEDONIA**								
T1	6.87	5.24	0.79	4.03	3.33	0.68
T2	5.95	3.58	0.54	5.85	4.21	0.86
**(5) SANS-ATTENTION**								
T1	4.33	2.85	0.43	2.53	2.16	0.44
T2	2.77	2.39	0.36	3.60	3.14	0.64
**SAS-TS**								
T1	4.83	3.78	0.57	2.83	2.60	0.53
T2	2.91	2.19	0.33	3.12	2.30	0.47

MI, Multiple Imputation; SANS-TS, Scale for the Assessment of Negative Symptoms Total Score; SANS-BA, Scale for the Assessment of Negative Symptoms Subscale Blunted Affect; SAS-TS, Simpson-Angus Scale Total Score; T1, Measurement Time 1 prior to the treatment period; T2, Measurement Time 2 after 10 weeks of treatment or waiting.

a n = 44.

b n = 24.

As shown in Table 4, despite randomization, mean baseline scores in all primary outcome variables were substantially higher in the treatment group than in the control group. *T*-tests revealed significant differences between the groups at the first point of measurement (T1) for the total score of negative symptoms [SANS-TS: *t*_(66)_ = −2.70, *p* < 0.01] and the subscores SANS-BA [*t*_(66)_ = −2.22, *p* < 0.05], SANS-Anhedonia [*t*_(66)_ = −2.37, *p* < 0.05] and SANS-Attention [*t*_(66)_ = −2.70, *p* < 0.01]. The differences disappeared at the second point of measurement (T2).

Within an outlier-analysis for the two groups respectively, we only found outliers with comparably high total scores in the control group. Therefore, they could not have been the reason for significant higher mean baseline scores of the treatment group. Logistic regressions of baseline scores on demographic variables did not show significant results for any of the potential confounders. Thus, baseline differences also did not stem from differences in any of the confounding demographic variables.

In the treatment group, however, we identified three participants, who had high SANS baseline scores compared to the entire sample. All of them were diagnosed with a residual condition of schizophrenia (ICD-10: F20.5). In order to rule out pseudo-effects produced by strong changes of comparably severe impaired patients we analyzed individual change scores of the three participants separately. Symptom reduction varied from 17 to 68.57%, unsystematically de- or exceeding the mean symptom reduction of 20.65% (see next paragraph on changes in severity of overall negative symptoms). Changes in symptom severity of the three most strongly impaired participants therefore did not systematically bias symptom reduction in the treatment group.

#### Changes in severity of overall negative symptoms

**Figure 2:**
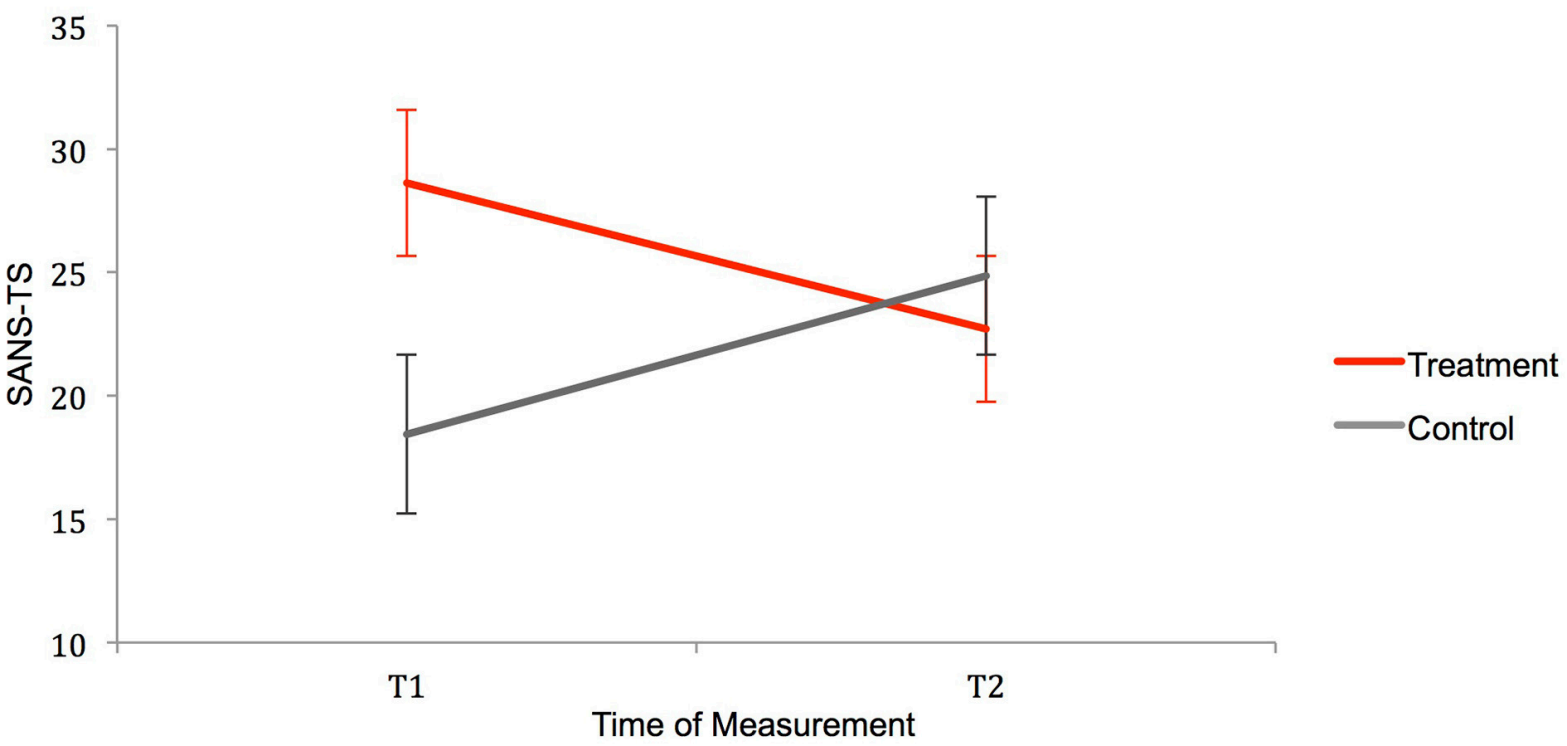
**Interaction of the factors Group and Time for the variable SANS-TS**. Error bars represent standard errors. Contrasts comparing SANS-TS of treatment and control group independently for the two testing times, reveal significant differences between the groups at T1, which even out at T2. Contrasts analyzing treatment and control group over time show an insignificant increase of negative symptoms in the control group and a significant reduction of negative symptoms in the treatment group. SANS-TS, Scale for the Assessment of Negative Symptoms Total Score; T1/T2, Measuring Time 1/Measuring Time 2.

Detailed results of the Mixed Model ANCOVA of the SANS total score (SANS-TS) are presented in Table 5 and Figure 2. The covariate SAS-CS, representing the amount of extrapyramidal side effects of participants, was significantly related to the amount of overall negative symptoms. It was therefore considered necessary to take SAS-CS into account as a confounding variable in all following analyses. Controlling for extrapyramidal side effects, the ANCOVA of patient's total scores revealed a highly significant effect of the treatment on the severity of overall negative symptoms: While the main effects of the two factors *Group* and *Time* were not significant, their interaction was significant at a level of 0.01. Contrasts, analyzing the two groups over time, displayed a non-significant increase of overall negative symptoms in the control group and a significant reduction of overall negative symptoms in the treatment group (see Table 6). Effect sizes of the interaction effect as well as the reduction in the treatment group depict moderate and therefore clinically substantial effects. Mean symptom reduction of the treatment group accounts for about 20.65% of the baseline symptom score. When the ANCOVA of SANS-TS was repeated with baseline scores as additional covariates, changes in overall negative symptom severity remained significant at a significance level of 0.05 [*F*_(1, 60.01)_ = 4.73, *p* = 0.03, *r* = 0.27], notwithstanding a Bonferroni correction for multiple testing.

**Table 5:** **Mixed model ANCOVA for SANS-TS with SAS-CS as covariate**.

**Effect**	***F***	***df***		***p***	***r***
		**Effect**	**Residual^*a*^**		
Group	0.77	1	61.73	0.38	0.13
Time	0.02	1	61.89	0.89	0.02
Group × Time	11.51	1	62.99	0.00^**^	**0.39**
SAS-CS	8.82	1	59.19	0.00^**^	0.20

Significant differences after Bonferroni correction are presented in bold. SAS-CS, Simpson Angus Scale Composite Score as a measure of overall extrapyramidal side effects; r, Pearson's r as a measure of the effect size for the comparison of two groups.

a Degrees of Freedom of the residual are adjusted downwards for each effect individually when analyzing multiply imputed data sets to take into account extra uncertainty due to missing data. For detailed formulas see van Ginkel (2014).

** p < 0.01.

**Table 6:** **Contrasts for treatment and control group over time**.

**Group**	**Variable_*Pair*_**	**MD**	**59% CI of** ***MD***		***t***	***df***	***r***
			**LL**	**UL**			
Treatment^a^	SANS-TS_T1−T2_	5.91	1.93	9.90	2.91^**^	43	**0.41**
(1)	SANS-BA_T1−T2_	2.07	0.35	3.80	2.36^*^	43	**0.34**
(2)	SANS-Alogia_T1−T2_	0.65	−0.46	1.75	1.15	43	0.17
(3)	SANS-Abulia_T1−T2_	1.05	−0.02	2.12	1.93	43	0.28
(4)	SANS-Anhedonia_T1−T2_	0.92	−0.47	2.31	1.30	43	0.19
(5)	SANS-Attention_T1−T2_	1.56	0.55	2.58	3.02^**^	43	**0.42**
Control^b^	SANS-TS_T1−T2_	−6.42	−12.78	−0.06	−1.98	23	0.38
(1)	SANS-BA_T1−T2_	−2.36	−5.83	1.10	−1.34	23	0.27
(2)	SANS-Alogia_T1−T2_	−0.61	−1.89	0.68	−0.93	23	0.19
(3)	SANS-Abulia_T1−T2_	−0.58	−2.20	1.04	−0.70	23	0.14
(4)	SANS-Anhedonia_T1−T2_	−1.82	−3.58	−0.05	−2.01^*^	23	0.38
(5)	SANS-Attention_T1−T2_	−1.07	−2.52	0.38	−1.44	23	0.29

Significant differences after Bonferroni correction are presented in bold. SANS-TS, Scale for the Assessment of Negative Symptoms Total Score; SANS BA, Scale for the Assessment of Negative Symptoms Subscale Blunted Affect; T1, Measurement Time 1 prior to the treatment period; T2, Measurement Time 2 after 10 weeks of treatment or waiting; r, Pearson's r as a measure of the effect size for the comparison of two groups. MD, Mean Difference; LL, lower limit; UL, upper limit.

a n = 47.

b ^b^n = 24.

* *p < 0.05*;

** p < 0.01.

#### Changes in severity of specific subtypes of negative symptoms

Results of the Mixed Model ANCOVAs of SANS subscores are shown in Table 7.

**Table 7:** **Mixed model ANCOVA for SANS sub-scores with SAS-CS as covariate**.

**Effect**	***F***	**df**		***P***	***r***
		**Effect**	**Residual^a^**		
**(1) SANS-BA**					
Group	0.74	1	61.26	0.39	0.11
Time	0.03	1	61.68	0.87	0.02
Group × Time	6.22	1	59.08	0.02^*^	0.31
SAS-CS	3.66	1	57.98	0.06	0.24
**(2) SANS-ALOGIA**					
Group	0.81	1	59.52	0.37	0.11
Time	0.00	1	59.81	0.96	0.00
Group × Time	1.94	1	59.46	0.17	0.18
SAS-CS	4.92	1	57.01	0.03^*^	0.28
**(3) SANS-ABULIA**					
Group	0.40	1	59.58	0.53	0.08
Time	0.25	1	59.34	0.62	0.06
Group × Time	2.96	1	60.02	0.09	0.22
SAS-CS	2.74	1	58.25	0.10	0.21
**(4) SANS-ANHEDONIA**					
Group	1.17	1	60.56	0.28	0.05
Time	0.59	1	60.41	0.45	0.10
Group × Time	5.47	1	59.10	0.02^*^	0.29
SAS-CS	8.34	1	57.15	0.01^*^	0.36
**(5) SANS-ATTENTION**					
Group	0.30	1	58.58	0.59	0.07
Time	0.30	1	53.52	0.59	0.07
Group × Time	9.14	1	59.81	0.00^**^	**0.36**
SAS-CS	4.03	1	53.54	0.05	0.26

Significant differences after Bonferroni correction are presented in bold. SAS-CS, Simpson Angus Scale Composite Score as a measure of overall extrapyramidal side effects; r, Pearson's r as a measure of the effect size for the comparison of two groups.

a Degrees of Freedom of the residual are adjusted downwards for each effect individually when analyzing multiply imputed data sets to take into account extra uncertainty due to missing data. For detailed formulas see van Ginkel (2014).

* *p < 0.05*;

** p < 0.01.

Controlling for side effects of medication, the ANCOVAs led to significant interaction effects for the subscores (1) SANS-BA, (4) SANS-Anhedonia, and (5) SANS-Attention. Only SANS-Attention withstood Bonferroni correction, being significant at a significance level of 0.01. Contrasts analyzing the change in subtypes of negative symptoms over time led to significant results for the same subscores. While for SANS-BA and SANS-Attention there was a significant reduction in the treatment group and no significant increase in the control group, for SANS-Anhedonia the pattern was reversed (See Table 6). Furthermore, effect sizes of severity reduction in SANS-BA and SANS-Attention were moderate. We can therefore only speak of a treatment effect on negative symptoms summarized in the subscores (1) Blunted Affect and (5) Attention, with subscore 5 being strongly or definitely affected. When ANCOVA was repeated for the two significantly reduced subscales with baseline scores as additional covariates diverging changes in treatment and control group did not result significant any more.

## Discussion

Following previous studies by Röhricht et al. (Röhricht and Priebe, 2006; Röhricht et al., 2009, 2011), the present randomized controlled trial aimed to investigate effects of BPT/DMT on negative symptoms in schizophrenia using an increased sample size, several therapists as well as different medical sites.

### Changes of overall symptom severity (hypothesis a)

As anticipated, BPT/DMT significantly reduced overall negative symptom severity in patients with schizophrenia when given in addition to TAU. Because antipsychotic medication remained stable during the assessment period and extrapyramidal side effects were controlled for in the analyses, the improvement found was independent of any change in positive symptoms or side effects. Resulting moderate effect sizes as well as the mean symptom reduction of 20.65% are in line with previous empirical findings of Röhricht and Priebe (2006), who found a mean symptom reduction of 20–25%. These symptom changes can be regarded as clinically substantial, when applying cut-off criteria of Levene et al. (Rector et al., 2003; Levine and Leucht, 2013).

Compared to results from studies on the efficacy of atypical antipsychotics, effect sizes and symptom reduction scores of the present study are encouragingly high: Chakos et al. found mean negative symptom reductions ranging between 3 and 15% for the respective antipsychotic agent (Chakos et al., 2001) and Leucht and colleagues report small to medium effect sizes varying between 0.09 and 0.32 (Leucht et al., 1999, 2009). However, none of the reviewed studies investigated negative symptoms separately. Improvements in negative symptoms therefore might have been secondary to reduced side effects of atypical neuroleptics or changes in positive symptoms, a concern that—as stated above—does not apply to the results of this study. Compared to recent attempts of treating negative symptoms with additional cognitive-behavioral therapy (CBT), effects of the present study remain promising. After reviewing more than twenty studies, Elis et al. (2013) emphasize the general efficacy of additional CBT regarding negative symptoms, with effect sizes ranging from small to large. The heterogeneity of the interventions' format and length as well as unspecified effect sizes, however, impeded the computation of a mean effect size and the drawing of interpretive conclusions. Moreover, in recent literature, adjunctive therapy with antidepressants has gained center stage for the reduction of negative symptoms. Singh et al. reviewed 23 trials and found moderate effects of treatment with antidepressants when given in addition to the usual treatment with antipsychotics (Singh et al., 2010).

### Changes in severity of symptom subtypes (hypothesis b)

As for the effect of BPT/DMT on specific negative symptoms, in addition to expectations, manualized movement therapy did not only have a reducing impact on the severity of blunted affect, but also on the severity of deficits in attention. In fact, only the effect on attention deficits was strong enough to withstand Bonferroni correction.

In the light of phenomenological concepts, which consider schizophrenia as a fundamental *disembodiment*, this finding very much supports the application of embodied therapies. Both blunted affect as well as attention deficits can be ascribed to an initial loss of embodied self-awareness. While blunted affect might arise from a subsequent alienation of somatosensory perception and its link to emotional and motivational context, attention deficits might be caused by the fragmentation of meaningful thought and action units and the resulting hyperreflexive awareness toward usually tacit aspects of everyday life: Limited attentional resources are consumed by the constant compensation for the disautomation of habitual bodily actions. By redirecting attention and mindfulness to the body and its connection to the self, BPT/DMT intervenes at the likely foundation of the schizophrenic illness and consequently of the respective negative symptoms. Furthermore, the attention subscale of the SANS particularly examines social attention. The specifically designed group setting of BPT/DMT interventions with its focus on the facilitation of emotional group interactions might be especially effective in promoting attention toward bodily mediated emotions of others and their impact on the self.

The effect of movement therapy on anhedonia, which was found in the respective ANCOVA, can statistically be considered a pseudo effect: As seen in the account of the contrasts, the inability to experience pleasure worsened significantly in the control group but did not improve significantly in the treatment group. However, similar to all other subscales, anhedonia symptom change in the treatment group had an encouraging tendency into the expected direction. This goes hand in hand with recent investigations on factors of effectiveness of embodied arts therapies. Locating further creative arts therapies, such as dance, drama, music, and art therapy within in the embodiment paradigm, Koch (2015) considers *hedonism* or *play* as one of the most important characteristics and catalysts of change in embodied therapies. By surrendering oneself to a playful, improvisational and non-judgemental mode when confronted with musical instruments, art materials or dance music, patients can experience a shared joy and thereby reactivate their functional indulgence or pleasure (German: “Funktionslust”), creativity (“me as a creator”) and self-efficacy (“I am in control”). Because this works best when basic inhibitory self-regulation is re-established by the appropriate dosage of an antipsychotic agent, medical treatment and embodied therapies together may deliver the most adequate model for state-of-the-art schizophrenia treatment.

## Limitations and future directions

When working with populations with severe mental illnesses drop-out rates are a common issue. Unsurprisingly one major limitation of the present study is its high rate of missing data due to drop-outs of participants. MI, as used in this study and described above, is currently one of the most sophisticated methods to meet problems of missing data.

Moreover, despite randomization, baseline differences existed in the major study variables prior to the treatment. Block randomization, which was used to allocate patients to groups, is known for the possibility of generating groups, that are rarely comparable in terms of certain variables or covariates (Suresh, 2011). In the present study all patients with diagnoses belonging to the schizophrenia spectrum were included but patients were not matched according to their specific subtype of diagnosis (IDC-10: F20.x, F25.x). It could therefore be due to the block randomization method and its inherent inaccuracy that by chance all patients suffering from a severe residual condition of schizophrenia (F20.5) were allocated to the treatment condition. Nonetheless, overall treatment effects remain significant after controlling for baseline differences at a significance level of 0.05. It is therefore justifiable to speak of clinically substantial effects, which need replication with an appropriate randomization technique in order to withstand Bonferroni correction in future studies.

Furthermore, due to financial constraints and time limitations, we worked with a waiting control group. Thus, non-specific group or exercise effects in the treatment group cannot fully be ruled out. Yet, the fact that Röhricht and Priebe (2006), who administered supportive counseling to their control group, obtained very similar results, points to a justified attribution of the effect to movement therapy. Future studies should pay attention to the specific features of BPT/DMT, distinguishing it from other group or physical exercise therapies. Such features are for example (a) the focus on body experience at a cognitive and emotional level, (b) the facilitation of emotional group interactions, and (c) the link between movement and emotion (Priebe et al., 2013). To reduce non-specific effects of non-emotional group interactions, therapist attention, and physical activity, an active control condition, such as pilates or moderate sports therapy, is recommended.

Other than that, as stated above, the structure and relationship of negative symptom domains are a point of constant debate. The SANS, although one of the most widely used negative symptom rating scales, is criticized by scholars and practitioners (Blanchard et al., 2011; Rabany et al., 2011) because it is supposed to include items not considered to belong to the negative symptom construct and fails to take into account subjective experiences of patients (Sass and Parnas, 2003; Kirkpatrick et al., 2006). A major challenge for the development of efficacious interventions is the valid and reliable assessment of negative symptoms. For the purpose of instrument development the negative symptom domains need to be clearly defined.

In a data-driven iterative process the Collaboration to Advance Negative Symptom Assessment in Schizophrenia (CANSAS) study has put a lot of effort into constructing a next-generation negative symptom scale: The Clinical Assessment Interview for Negative Symptoms (CAINS; Kring et al., 2013). In 2012, when the TESIS study started, the new assessment tool unfortunately was not available yet. Future treatment development or efficacy studies should consider using the CAINS as an additional assessment tool.

As part of the larger TESIS study, which the present trial was embedded in, other clinical as well as psychosocial and neurological variables, such as the overall severity of psychopathological symptoms, psychological functioning, body experience, ego pathology, ego demarcation, quality of life, empathy, or social sensitivity (Theory of Mind), and neurological soft signs (NSS) were assessed for the same patients (Hirjak et al., 2014, 2015). It will be illuminating to analyze the interaction of changes in negative symptoms with those manifold constructs to see whether and to what extent reduced negative symptoms are accompanied by, for example, improved social contact or better quality of life. Lastly, on the basis of preliminary findings from a few empirical studies, Röhricht (2009) states that the impact of BPT/DMT therapeutic processes spans across various domains targeted by conventional therapies, such as cognitive reconstruction in CBT or insight-oriented processes in psychodynamic therapies. Future analyses may explore in depth the therapeutic mechanisms involved in BPT/DMT treatment processes.

## Conclusion

The present study contributes to the encouraging evidence for the use of embodied therapies, specifically BPT/DMT, in the treatment of schizophrenia. It successfully replicates positive effects of movement therapy on negative symptom severity, which match or even surmount the efficacy of conventional pharmacological and psychological treatment. Although some scholars and many practitioners argue that merely quantitative research is not capable of capturing the various mechanisms of change of embodied therapies, such as the therapeutic relationship, aesthetics, or creativity (Chace, 1957; Koch et al., 2014), further evidence-based research is needed to establish embodiment-based interventions in the common treatment of severe mental disorders. On this road, the embodiment paradigm provides a broad theory framework and extensive opportunities for body-oriented and non-verbal disciplines (BPT, DMT, music-, art-, and drama-therapy) with interdisciplinary bridges to phenomenology, psychology, psychiatry, and cognitive neuroscience to support our claim that both medication and embodied therapies together deliver the most appropriate form of present state schizophrenia treatment.

## Author contributions

All authors contributed substantially to this work. LM (the corresponding author) organized, reduced and analyzed the data and wrote the article, SK and DH supervised and supported the data collection, SK oversaw the analysis, TF and SK designed the study, TF was the primary investigator under whose supervision the study was conducted and acquired the funding. All authors contributed to the writing process, they discussed the results and implications and commented on the manuscript at all stages.

## Funding

The trial constituted the randomized controlled part of a larger study, conducted at the Department of General Psychiatry in Heidelberg, Germany, as part of the EU-project “Toward an Embodied Science of Intersubjectivity (TESIS: https://tesisnetwork.wordpress.com/).” It was approved by the local ethics committee of the Medical Faculty of the University of Heidelberg, registered with DRKS (German Clinical Trials Register: DRKS00009828, http://apps.who.int/trialsearch/) and funded by the TESIS project as well as private sponsors.

### Conflict of interest statement

The authors declare that the research was conducted in the absence of any commercial or financial relationships that could be construed as a potential conflict of interest.
