# Supplementary material for: Overcoming Disembodiment: The Effect of Movement Therapy on Negative Symptoms in Schizophrenia—A Multicenter Randomized Controlled Trial
Source: Front Psychol. 2016 Mar 31;7:483. doi: 10.3389/fpsyg.2016.00483 (PMC4815039; doi:10.3389/fpsyg.2016.00483)
Supplement: Supplementary file 1 [file Presentation1.PDF]

Supplementary Material

Overcoming Disembodiment: The Effect of Movement Therapy on Negative Symptoms in Schizophrenia - A Multicenter Randomized Controlled Trial

Lily Martin[1*], Sabine C. Koch[2], Dusan Hirjak[3], Thomas Fuchs[3]

[1*]*Dept of Educational Science and Psychology, Free University Berlin, Berlin, Germany*

[2]*Dept of Dance and Movement Therapy, School of Therapeutic Sciences, SRH University Heidelberg, Heidelberg, Germany*

[3]*Dept of General Psychiatry, Centre for Psychosocial Medicine, Academic Medical Center, University of Heidelberg, Heidelberg, Germany*

1 Supplementary Information on the TESIS Project

In the following section the official flyer of the TESIS research project is displayed.

Das TESIS-Projekt

Durchführung

- In unserem 10-wöchigen Programm werden ausgebildete Bewegungstherapeuten/innen mit Teilnehmern/innen ausgewählter Einrichtungen die Therapie im Rhein-Neckar Raum durchführen. Innerhalb dieses Angebots können die Teilneh-mer/innen in einem geschützten und vertrauten Rahmen Erfahrungen mit ihrem Körper und ihrer Bewegungs- und Ausdrucksfähigkeit im Kontakt mit anderen machen. Vor und nach der Gruppe werden in 1-2 Treffen diagnostische Fragen geklärt.*1
- Psychometrische Diagnostik (SAPS, SANS, BPRS, SCS, EASE, NSS, CEEQ, FP-Test und andere)*2
- Multimodale Bildgebung des Gehirns mittels struktureller und funktioneller Magnetresonanztomographie mit Befund
- Aufwandsentschädigung für den/die Patienten/in

*1 *Aus der bisherigen Anwendung der Bewegungstherapie sind zahlreiche Vorteile für die Teilnehmer/innen bekannt:*
- Steigerung der sozialen Kompetenz
- Steigerung des Einfühlungsvermögen
- Bessere Wahrnehmung des eigenen Körpers
- Bessere Wahrnehmung anderer Person
- Verbesserung der allgemeinen Befindlichkeit

*2 *Wir stellen die Befunde der neuropsychologischen Untersuchung und der strukturellen Bildgebung bei Einwilligung des Patienten gerne auch dem/der ambulanten Behandler/in zur Verfügung.*

Ansprechpartner

Für weitere Informationen bezüglich unserer Studie wenden Sie sich telefonisch oder per Email an:

Dipl.-Psych. Laura Galbusera
Klinik für Allgemeine Psychiatrie
Telefon: 06221-56-37411
Laura.galbusera@med.uni-heidelberg.de

Dr. med. Dusan Hirjak
Klinik für Allgemeine Psychiatrie
Telefon: 06221-56-37539
Dusan.hirjak@med.uni-heidelberg.de

Laura Mehl, B.A. Psych.
Psychologisches Institut Heidelberg
Universität Heidelberg
Telefon: 0176-83259919
Laura-mehlis@web.de

Studienleitung:
Prof. Dr. med. Dr. phil. T. Fuchs

Stellvertretung:
Prof. Dr. phil. S. Koch

Sekretariat:
Waltraud Werkmann
Klinik für Allgemeine Psychiatrie
Voßstraße 2
69115 Heidelberg
Telefon: 06221-56-7275
Waltraud.werkmann@med.uni-heidelberg.de

ID22672 Unternehmenskommunikation/Medienzentrum Universitätsklinikum Heidelberg, Bildquelle: www.istockphoto.com

UniversitätsKlinikum Heidelberg

Informationen für Interessierte

Das TESIS-Projekt

der Klinik für Allgemeine Psychiatrie am Zentrum für Psychosoziale Medizin Universitätsklinikum Heidelberg

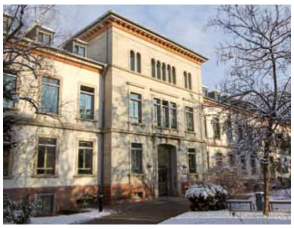

Das TESIS-Projekt

Theoretischer Hintergrund

Aus phänomenologischer Sicht lässt sich der Autismus als eine grundlegende Abweichung der Wahrnehmung des eigenen Selbst beschreiben, in Verbindung mit Auffälligkeiten in der sozialen Interaktion. Dazu gehört insbesondere eine verminderte Fähigkeit, Empathie zu empfinden, also sich in den emotionalen Zustand einer anderen Person einzufühlen. Empathie ist nicht nur von hoher Bedeutung für unsere Gesellschaft, sondern auch wichtig als Basis für die Entwicklung eines eigenen Selbstverständnisses.

Die Schizophrenie wird als eine fundamentale Störung der Verkörperung des eigenen Selbst in Verbindung mit Beeinträchtigungen der zwischenleiblichen Kommunikation betrachtet. Manche Autoren beschreiben eine mangelnde emotionale Resonanzfähigkeit schizophrener Personen als Grundlage des reduzierten Einfühlungsvermögens.

Die Resonanzfähigkeit, die bei beiden klinischen Bildern ein zentraler Aspekt zu sein scheint, gilt es deshalb therapeutisch zu stärken. Folgen wir Merleau-Ponty (1966), so liegt die Grundlage der Resonanzfähigkeit in der leiblichen Kinästhetik. Leibarbeit und Bewegungstherapie sind auf dieser Grundlage in besonderer Weise geeignet, die innerund zwischenleibliche Resonanzfähigkeit eines Menschen wiederherzustellen oder zumindest zu verbessern.

Ziele

Unsere Studie ist in das Marie Curie ITN-Projekt TESIS („Towards an Embodied Science of InterSubjectivity"; 2011-2015) eingebettet und wird mit einer Population von 80 Patienten mit einer Störung aus dem schizophrenen Formenkreis und 80 Personen mit einer Autismus-Spektrums-Störung durchgeführt.

Zur Klärung der Frage, inwiefern Ansätze aus der Embodiment-Forschung und direkt darauf aufbauende therapeutische Methoden im Vergleich zu vorherrschenden kognitiven Ansätzen besser geeignet sind, führen wir insgesamt 3 Substudien durch. Das Ziel ist einerseits die Psychopathologie dieser beiden Störungen zu erklären und andererseits die vorliegenden Symptome zu behandeln.

Dazu dient zum einen der RCT zur Wirkung von Bewegungstherapie (tanz- und bewegungstherapeutische Spiegelungstechniken im Rahmen breiterer body-oriented psychological therapy; BOPT) bei schizophrenen und autistischen Patienten und zum anderen das phänomenologische EASE-Interview zur Erfassung der Verkörperungsaspekte der jeweiligen Psychopathologie für jede der beiden Hauptzielgruppen. Mit Hilfe der strukturellen und funktionellen Bildgebung (sMRT und fMRT) werden die zerebralen Korrelate der diskreten neurologischen Zeichen („Neurological Soft Signs" - NSS) untersucht und zu den übrigen Resultaten in Beziehung gesetzt.

An wen richtet sich unser bewegungstherapeutisches Angebot?

An Personen mit:

- der Diagnose einer Erkrankung aus dem schizophrenen Formenkreis (ICD 10 F20-29) – außer F23 Akute vorübergehende psychotische Störungen
- der Diagnose einer Autismus-Spektrum-Störung (ICD 10 F84.0, F84.1, F84.5, F84.9)
- einem Alter von 14 bis 65 Jahren
- einer recent-onset Schizophrenie (psychotische Erstmanifestation innerhalb der letzten 24 Monate – für den Einschluss in die phänomenologischen Interview-Studie)
- Vollremission psychotischer Symptome
- Einwilligungsfähigkeit und informierter Einwilligung

Ausschlusskriterien:

- Neurologische oder internistische Erkrankung oder ein Schädel-Hirn-Trauma
- Bestehender schädlicher Gebrauch oder Abhängigkeit von psychotropen Substanzen
- Einwilligungsunfähigkeit
- Intelligenzquotient < 70

2 Supplementary Information on the research procedure

In the following section the applied assessment instruments are displayed.

2.1 The German Version of the Scale for the Assessment of Negative Symptoms (SANS) to capture negative symptom severity

Scale for Assessment of Negative Symptoms – **SANS**

**I. AFFEKTVERFLACHUNG UND AFFEKTSTARRHEIT**<br>Affektverflachung und Affektstarrheit manifestiert sich als eine charakteristische *Verarmung der emotionalen Ausdrucksfähigkeit,* der *Reaktionsfähigkeit* und des *Fühlens.*<br>Zur Beurteilung der nachfolgenden Items sollten normalerweise affektbesetzte Themen zur Sprache gebracht werden.		
**1. Starrer Gesichtsausdruck**<br>Das Gesicht des Patienten erscheint hölzern, mechanisch, gefroren. Der Gesichtsausdruck wechselt nicht oder weniger als der gefühlsmäßige Inhalt des Gesprächs erwarten lässt. Da Neuroleptika teilweise diesen Effekt nachahmen, sollte der Beurteiler sorgfältig festhalten, ob der Patient eine neuroleptische Medikation erhält, nicht aber versuchen, die Beurteilung dieses Symptoms diesem Neuroleptika bedingten Effekt entsprechend anzupassen.	**0**	= normal
	**1**	= Der pathologische Charakter des beobachteten Phänomens ist zweifelhaft
	**2**	= Diskret pathologische Ausprägung
	**3**	= Pathologische Ausprägung erkennbar
	**4**	= Pathologische Ausprägung für jeden Beobachter erkennbar
	**5**	= Gravierender Ausprägungsgrad
**2. Verminderte Spontanbewegungen**<br>Der Patient sitzt während des Gesprächs ruhig da und zeigt wenig oder keine Spontanbewegungen. Er wechselt seine Sitzstellung nicht, bewegt Beine oder Hände nicht oder weniger als normalerweise erwartet wurde.	**0**	= normal
	**1**	= Der pathologische Charakter des beobachteten Phänomens ist zweifelhaft
	**2**	= Diskret pathologische Ausprägung
	**3**	= Pathologische Ausprägung erkennbar
	**4**	= Pathologische Ausprägung für jeden Beobachter erkennbar
	**5**	= Gravierender Ausprägungsgrad
**3. Armut der Ausdrucksbewegung**<br>Der Patient verwendet seinen Körper nicht als Hilfsmittel um sich auszudrücken, wie z.B. Handbewegungen, sich vorlehnen im Stuhl oder entspanntes Zurücklehnen. Dies ist zusätzlich zu verminderten Spontanbewegungen zu beurteilen.	**0**	= normal
	**1**	= Der pathologische Charakter des beobachteten Phänomens ist zweifelhaft
	**2**	= Diskret pathologische Ausprägung
	**3**	= Pathologische Ausprägung erkennbar
	**4**	= Pathologische Ausprägung für jeden Beobachter erkennbar
	**5**	= Gravierender Ausprägungsgrad

Scale for Assessment of Negative Symptoms – **SANS**

**4. Geringer Augenkontakt**<br><br>Der Patient vermeidet Blickkontakt während des Gesprächs oder die Augenpartie lässt keine Expression erkennen. Er scheint ins Weite zu starren, selbst dann, wenn er spricht.	**0**	= normal
	**1**	= Der pathologische Charakter des beobachteten Phänomens ist zweifelhaft
	**2**	= Diskret pathologische Ausprägung
	**3**	= Pathologische Ausprägung erkennbar
	**4**	= Pathologische Ausprägung für jeden Beobachter erkennbar
	**5**	= Gravierender Ausprägungsgrad
**5. Fehlende affektive Reaktionsfähigkeit (Affektstarrheit)**<br><br>Der Patient reagiert nicht, oder nicht ausreichend auf Gesprächsinhalte, die normalerweise mit heiterem oder traurigem Affekt beantwortet werden. Unfähigkeit zu lachen oder zu lächeln, wenn evoziert. Das kann geprüft werden, indem man lacht oder in einer Weise Witze macht, dass man von einem normalen Menschen ein Lachen hervorrufen würde. Der Prüfer kann ebenso fragen, „ Haben Sie vergessen zu lachen?“, während er selbst lächelt.	**0**	= normal
	**1**	= Der pathologische Charakter des beobachteten Phänomens ist zweifelhaft
	**2**	= Diskret pathologische Ausprägung
	**3**	= Pathologische Ausprägung erkennbar
	**4**	= Pathologische Ausprägung für jeden Beobachter erkennbar
	**5**	= Gravierender Ausprägungsgrad
**6. Mangel an vokaler Ausdrucksfähigkeit (monotone Sprache)**<br><br>Beim Sprechen gelingt es dem Patienten nicht, ein normales Betonungsschema der Stimme zu zeigen. Wichtige Worte sind durch Wechsel in Tonhöhe und Tonstärke charakterisierbar. Keine Modulation der Lautstärke bei vertraulichen oder allgemein interessierenden Gesprächsinhalten.	**0**	= normal
	**1**	= Der pathologische Charakter des beobachteten Phänomens ist zweifelhaft
	**2**	= Diskret pathologische Ausprägung
	**3**	= Pathologische Ausprägung erkennbar
	**4**	= Pathologische Ausprägung für jeden Beobachter erkennbar
	**5**	= Gravierender Ausprägungsgrad

Scale for Assessment of Negative Symptoms – **SANS**

**7. Globale Beurteilung der affektiven Verminderung**<br><br>Die Globalbeurteilung sollte sich auf den allgemeinen Schweregrad der Affektverflachung und Affektstarrheit beziehen. Besondere Bedeutung sollte Hauptmerkmalen, wie *mangelnde Auslenkbarkeit, Inadäquatheit* und die *allgemeine Verminderung der emotionalen Intensität* gegeben werden.	**0**	= normaler Affekt
	**1**	= fragliche affektive Verminderung
	**2**	= leichte affektive Verminderung
	**3**	= mäßige affektive Verminderung
	**4**	= deutliche affektive Verminderung
	**5**	= schwere Affektverflachung und Affektstarrheit
Subskalenwert I (Summe der Items 1 bis 7):		

II. ALOGIE UND PARALOGIE

Alogie beschreibt die Verarmung der Denkens und der Wahrnehmung, wie man sie bei schizophrenen Patienten findet. Der Denkprozess wirkt leer, verworren und langsam. Alogie manifestiert sich in einer stockenden, leeren Sprache (*Armut der Sprache*) oder in einer flüssigen, aber inhaltsleeren Sprache (*Verarmung des Gesprächsinhaltes*) und/oder *erhöhter Antwortlatenz.*

**8. Verarmung der Sprechweise**<br><br>Es handelt sich um eine Verminderung in der Menge des spontanen Gesprächs, so dass die Antworten zu Fragen dazu tendieren, kurz, konkret und unausgearbeitet zu sein. Ungefragte zusätzliche Informationen werden spärlich geliefert. Beispiel: Frage: „Wie viele Kinder haben Sie?“ Antwort: „2, ein Bub und ein Mädchen, das Mädchen ist 13 und der Bub ist 10.“ – „2“ wäre alles was zur Beantwortung erforderlich ist und der Rest ist zusätzliche Information. Antworten können auch einsilbig sein. Manche Fragen können undbeantwortet bleiben. Wenn der Interviewer mit dieser Sprechweise konfrontiert wird, kann er sich oft dabei ertappen, nachzufragen, um den Patienten in der Ausarbeitung der Antworten zu ermuntern. Um diesen Befund ans Licht zu bringen, muss der Prüfer dem Patienten ausreichend Zeit zur Beantwortung zur Verfügung stellen.	**0**	= keine Verarmung; eine substanzielle und entsprechende Anzahl von Antworten auf Fragen enthalten zusätzliche Informationen
	**1**	= fragliche Verarmung
	**2**	= leichte Verarmung; gelegentliche Antworten enthalten keine ausführliche Informationen, selbst wenn dies angebracht ist.
	**3**	= mäßige Verarmung der Sprache. Einige Antworten enthalten eine geeignete zusätzliche Information, viele Antworten sind einsilbig und sehr kurz (z.B. „Ja“, „Nein“, „Vielleicht“, „Letzte Woche“ etc.)
	**4**	= deutliche Sprachverarmung; Antworten sind selten länger als einige Worte
	**5**	= schwere Verarmung; Patient sagt sehr wenig und gelegentlich gelingt es ihm nicht zu antworten

27

Scale for Assessment of Negative Symptoms – **SANS**

<table>
<tr><td rowspan="6">9. Verarmung des Gesprächsgehalts (Gesprächsinhalt)

Obwohl die Antworten adäquat sind, enthalten sie nur wenig Information. Die Sprache tendiert zu Unbestimmtheit, oft überabstrakt und überkonkret, wiederholend und stereotyp. Der Interviewer kann diesen Befund durch die Beobachtung erkennen, nämlich dass der Patient zwar lange gesprochen hat, aber ohne adäquate Informationen zur Beantwortung der Fragen. Andererseits kann der Patient genug Informationen liefern, doch er benötigt dazu eine übermäßige Anzahl von Worten, obwohl die Antwort in ein bis zwei Sätzen subsummiert werden könnte. Manchmal kann man die Sprechweise auch als „leeres philosophieren“ charakterisieren. Ausschluss: Unterschied zur Weitschweifigkeit, das der weitschweifige, abschweifende Patient dazu tendiert, eine Fülle von Detailinformationen zu liefern.</td><td>0</td><td>= keine Verarmung</td></tr>
<tr><td>1</td><td>= fragliche Verarmung</td></tr>
<tr><td>2</td><td>= leichte Verarmung; gelegentliche Antworten sind unklar, um begreiflich zu sein oder die Antwort könnte merklich gestrafft werden</td></tr>
<tr><td>3</td><td>= mäßige Verarmung; häufig sind Antworten unklar oder könnten merklich gestrafft werden, um das Gespräch um mind. ¼ zu verkürzen</td></tr>
<tr><td>4</td><td>= merkliche Verarmung; mindestens die Hälfte des Gesprächs ist davon betroffen</td></tr>
<tr><td>5</td><td>= schwer; nahezu das gesamte Gespräch ist unklar und /oder unfassbar</td></tr>
<tr><td rowspan="6">10. Sperrungen

Unterbrechungen des Sprachduktus, bevor ein Gedanke oder eine Idee vollendet wurde. Nach einer gewissen Schweigezeit, welche von wenigen Sekunden bis zu Minuten dauern kann, gibt der Patient an, dass er nicht mehr abrufen kann, was er vorhatte zu sagen oder was er meinte zu sagen. Sperrungen sollten nur dann als vorhanden beurteilt werden, wenn der Patient freiwillig das „Verlieren seiner Gedanken“ beschreibt, oder wenn der Patient auf die Frage des Interviewers angibt, das dies der Grund für seine Pause war.</td><td>0</td><td>= keine Sperrungen</td></tr>
<tr><td>1</td><td>= fraglich</td></tr>
<tr><td>2</td><td>= leichte Sperrungen; einmaliges Auftreten während 15 Minuten</td></tr>
<tr><td>3</td><td>= mäßige Sperrungen; zweimaliges Auftreten während 15 Minuten</td></tr>
<tr><td>4</td><td>= merkliche Sperrungen; dreimaliges Auftreten während 15 Minuten</td></tr>
<tr><td>5</td><td>= schwere Sperrungen; mehr als dreimaliges Vorkommen</td></tr>
</table>

Scale for Assessment of Negative Symptoms – **SANS**

**11. Erhöhte Antwortlatenz**<br><br>Der Patient braucht unverhältnismäßig lange, um Fragen zu beantworten. Bei Nachfragen zeigt sich jedoch, dass der Patient die Frage aufgefasst hat, es ihm aber schwerfällt, seine Gedanken zu entwickeln	**0**	= nicht vorhanden
	**1**	= fraglich
	**2**	= manchmal Pausen kurz bevor der Patient antwortet
	**3**	= deutliche Pausen
	**4**	= ausgeprägte Verlängerung der Antwortlatenz
	**5**	= lange Pausen vor nahezu allen Antworten oder der Patient kann kaum antworten
**12. Globale Beurteilung der Alogie**<br><br>Da der Kernbefund von Alogie die Verarmung des Sprechens und des Gesprächsinhaltes sind, sollte die globale Beurteilung besonders diesem Umstand Rechnung tragen.	**0**	= keine Alogie
	**1**	= fragliche Alogie
	**2**	= leichte aber sichere Verarmung des Denkens
	**3**	= deutliche Verarmung des Denkens
	**4**	= ausgeprägte Verarmung; das Denken scheint meist verarmt
Subskalenwert II (Summe der Items 8 bis 12):		

**III. ABULIE – APATHIE**<br><br>Abulie manifestiert sich als ein charakteristischer *Mangel an Energie, Antrieb* und *Interesse.* Gelegentlich sind die Patienten von außen anregbar, bei Wegfall der Anregung erlischt der Antrieb jedoch rasch.		
**13. Pflege und Hygiene**<br><br>Der Patient widmet seiner Pflege und Hygiene wenig Aufmerksamkeit, er muss zur Körperpflege angehalten werden.	**0**	= nicht vorhanden
	**1**	= fraglich
	**2**	= leichte, aber sichere Anzeichen von Nachlässigkeit
	**3**	= unordentliche Erscheinung; der Patient muss gelegentlich zur Körperpflege angehalten werden
	**4**	= deutlich unordentliche Erscheinung; der Patient muss regelmäßig zur Körperpflege angehalten werden
	**5**	= extrem unordentlich und ungepflegt

Scale for Assessment of Negative Symptoms – **SANS**

**14. Unstetigkeit in Beruf und Ausbildung**<br><br>Der Patient geht keiner geregelten Arbeit nach und /oder zeigt in dieser Richtung Verantwortungslosigkeit.<br>Bei *stationären Patienten*: der Patient muss zur Arbeits- und Beschäftigungstherapie angehalten werden oder weigert sich dorthin zu gehen.	**0**	= nicht vorhanden
	**1**	= fraglich
	**2**	= leichte Anzeichen von Unstetigkeit
	**3**	= mäßige Anzeichen; der Patient muss gelegentlich dazu angehalten werden
	**4**	= merkliche Anzeichen; der Patient muss regelmäßig dazu angehalten werden
	**5**	= dem Patienten gelingt es nicht, Arbeit oder Beschäftigung konsequent durchzuführen
**15. Körperliche Energielosigkeit**<br><br>Der Patient neigt zu körperlicher Trägheit. Er sitzt stundenlang im Stuhl und unternimmt keine spontanen Aktivitäten. Die meiste Zeit verbringt er mit passiven Tätigkeiten, wie z.B. Fernsehen, etc. oder er ist untätig.	**0**	= nicht vorhanden
	**1**	= fraglich
	**2**	= leichte Energielosigkeit
	**3**	= mäßig; der Patient muss gelegentlich zu Aktivitäten angehalten werden
	**4**	= der Patient muss regelmäßig zu Aktivitäten angehalten werden
	**5**	= der Patient verhält sich die meiste Zeit völlig passiv
**16. Globale Beurteilung von Willensschwäche - Apathie**<br><br>Die globale Beurteilung sollt den allgemeinen Schweregrad der Abulie-Symptomatik reflektieren, wobei vorhandenen, zu erwartenden Umständen, der soziale Status und der Herkunft der Patienten entsprochen werden sollte. Bei der Durchführung der Globalbeurteilung sollte ein starkes Gewicht zu ein oder zwei hervorstechenden Symptomen, wenn sie speziell zutreffen, gegeben werden.	**0**	= normal
	**1**	= Der pathologische Charakter des beobachteten Phänomens ist zweifelhaft
	**2**	= Diskret pathologische Ausprägung
	**3**	= Pathologische Ausprägung erkennbar
	**4**	= Pathologische Ausprägung für jeden Beobachter erkennbar
	**5**	= Gravierender Ausprägungsgrad
Subskalenwert III (Summe der Items 13 bis 16):		

Scale for Assessment of Negative Symptoms – **SANS**

IV. ANHEDONIE – UNGESELLIGKEIT

Dieser Symptomkomplex beinhaltet die Schwierigkeiten des Patienten Interesse und/oder Vergnügen zu empfinden. Dies kann sich in Interessenverlust an angenehmen Aktivitäten und/oder Unfähigkeit bei solchen Tätigkeiten Vergnügen zu empfinden, äußer und/oder als Desinteresse an sozialen Beziehungen verschiedenster Art

**17. Freizeitvergnügen und Aktivitäten**<br>Der Patient hat weniger oder keine Interessen, Aktivitäten oder Hobbys. Obwohl dieses Symptom schleichend oder langsam beginnen kann, wird man im Allgemeinen ein deutliches Desinteresse an früheren Aktivitätsniveaus bemerken.<br>Nicht beurteilt werden soll eine eventuelle schizoide Primärpersönlichkeit, die Desinteresse an sozialen Beziehungen zeigt.	**0**	= normal
	**1**	= Der pathologische Charakter des beobachteten Phänomens ist zweifelhaft
	**2**	= Diskret pathologische Ausprägung
	**3**	= Pathologische Ausprägung erkennbar
	**4**	= Pathologische Ausprägung für jeden Beobachter erkennbar
	**5**	= Gravierender Ausprägungsgrad
**18. Sexuelles Interesse**<br>Beurteilt werden soll das Interesse und die Bereitschaft sexuellen Kontakt aufzunehmen.	**0**	= normal
	**1**	= Der pathologische Charakter des beobachteten Phänomens ist zweifelhaft
	**2**	= Diskret pathologische Ausprägung
	**3**	= Pathologische Ausprägung erkennbar
	**4**	= Pathologische Ausprägung für jeden Beobachter erkennbar
	**5**	= Gravierender Ausprägungsgrad
**19. Fähigkeit, Intimität und Nähe zu fühlen**<br>Beurteilt werden soll die Fähigkeit, zu einer Kontaktperson eine persönliche Beziehung aufzubauen und aufrechtzuerhalten.	**0**	= normal
	**1**	= Der pathologische Charakter des beobachteten Phänomens ist zweifelhaft
	**2**	= Diskret pathologische Ausprägung
	**3**	= Pathologische Ausprägung erkennbar
	**4**	= Pathologische Ausprägung für jeden Beobachter erkennbar
	**5**	= Gravierender Ausprägungsgrad

Scale for Assessment of Negative Symptoms – **SANS**

**20. Verhalten zu Verwandten und Kollegen (Autismus)**<br><br>Beurteilt werden soll die Fähigkeit des Patienten, in seinem sozialen Umfeld Kontakte zu knüpfen. Autistische Patienten nehmen spontan keine Kontakte auf; wenn man sie dazu anregt reagieren sie entweder gereizt oder versinken bei Wegfall der Anregung in den alten Zustand.	**0**	= normal
	**1**	= Der pathologische Charakter des beobachteten Phänomens ist zweifelhaft
	**2**	= Diskret pathologische Ausprägung
	**3**	= Pathologische Ausprägung erkennbar
	**4**	= Pathologische Ausprägung für jeden Beobachter erkennbar
	**5**	= Gravierender Ausprägungsgrad
**21. Globale Beurteilung der Anhedonie - Ungeselligkeit**<br><br>Die Globalbeurteilung sollte den allgemeinen Schweregrad des Anhedonie-Ungeselligkeits-Komplexes reflektieren.	**0**	= normal
	**1**	= Der pathologische Charakter des beobachteten Phänomens ist zweifelhaft
	**2**	= Diskret pathologische Ausprägung
	**3**	= Pathologische Ausprägung erkennbar
	**4**	= Pathologische Ausprägung für jeden Beobachter erkennbar
	**5**	= Gravierender Ausprägungsgrad
Subskalenwert IV (Summe der Items 17 bis 21):		

V. AUFMERKSAMKEIT

Die Aufmerksamkeit ist bei Schizophrenen oft gestört. Der Patient ist nicht fähig, seine Aufmerksamkeit auf etwas Bestimmtes zu konzentrieren und/oder er ist lediglich fähig, sich sporadisch und sprunghaft zu konzentrieren.

**22. Soziale Unaufmerksamkeit**<br><br>Der Patient hat von sich aus kein Interesse an sozialen Aktivitäten. Diese Interessenlosigkeit kann sich u.a. in negativistischem Verhalten äußern und/oder Versuche an sozialen Aktivitäten teilzunehmen scheitern an der mangelnden Konzentrationsfähigkeit.	**0**	= normal
	**1**	= Der pathologische Charakter des beobachteten Phänomens ist zweifelhaft
	**2**	= Diskret pathologische Ausprägung
	**3**	= Pathologische Ausprägung erkennbar
	**4**	= Pathologische Ausprägung für jeden Beobachter erkennbar
	**5**	= Gravierender Ausprägungsgrad

Scale for Assessment of Negative Symptoms – **SANS**

**23. Unaufmerksamkeit während psychologischer Testung**<br><br>Bei einmaliger Anwendung der Skala sollte der Mini-Mental-Status-Test (MMST) durchgeführt werden; dieser Test kann nach ca. 3 bis 4 Wochen wiederholt werden:<br><br>Bei regelmäßiger Anwendung und zur Beurteilung des Therapieerfolges sollte folgendermaßen vorgegangen werden:<br><br>Wiederholung nach 1 Woche: 7 von 100 abziehen lassen.<br>Wiederholung nach 2 Wochen: 4-stellige Zahl rückwärts.<br>Wiederholung nach 3 Wochen:9 von 100 abziehen lassen.<br>Bei Untersuchungsende MMST	**0**	= kein Fehler
	**1**	= fraglich (Patient führt die Aufgabe in zögernder Weise aus und/oder macht einen Fehler, den er sofort korrigiert)
	**2**	= 1 Fehler
	**3**	= 2 Fehler
	**4**	= 3 Fehler
	**5**	= mehr als 3 Fehler
**24. Globale Beurteilung der Aufmerksamkeit**<br><br>Beurteilt werden soll die Aufmerksamkeit und Konzentrationsfähigkeit, wobei sowohl das klinische Bild wie die Ausführungen von Aufgaben in Betracht gezogen werden sollen.	**0**	= normal
	**1**	= Der pathologische Charakter des beobachteten Phänomens ist zweifelhaft
	**2**	= Diskret pathologische Ausprägung
	**3**	= Pathologische Ausprägung erkennbar
	**4**	= Pathologische Ausprägung für jeden Beobachter erkennbar
	**5**	= Gravierender Ausprägungsgrad

Subskalenwert V (Summe der Items 22 bis 24):	
Summary Score/Summe der Globalratings (Item 7,12,16,21 und 24):	
Composite Score/Gesamtskalenwert (Subskalenwert I,II,III,IV und V):	

2.2 The Simpson-Angus Scale (SAS) for the assessment of extrapyramidal side effects of antipsychotic medication

SIMPSON-ANGUS SCALE

(SAS)

SIMPSON-ANGUS SCALE (SAS)

1. GAIT:
The patient is examined as he walks into the examining room, his gait, the swing of his arms, his general posture, all form the basis for an overall score for this item. This is rated as follows:

**0** = Normal	☐
**1** = Diminution in swing while the patient is walking	☐
**2** = Marked diminution in swing with obvious rigidity in the arm	☐
**3** = Stiff gait with arms held rigidly before the abdomen	☐
**4** = Stopped shuffling gait with propulsion and retropulsion	☐

2. ARM DROPPING:
The patient and the examiner both raise their arms to shoulder height and let them fall to their sides. In a normal subject, a stout slap is heard as the arms hit the sides. In the patient with extreme Parkinson's syndrome, the arms fall very slowly:

**0** = Normal, free fall with loud slap and rebound	☐
**1** = Fall slowed slightly with less audible contact and little rebound	☐
**2** = Fall slowed, no rebound	☐
**3** = Marked slowing, no slap at all	☐
**4** = Arms fall as though against resistance; as though through glue	☐

SIMPSON-ANGUS SCALE (SAS)

**3. SHOULDER SHAKING:**<br>The subject's arms are bent at a right angle at the elbow and are taken one at a time by the examiner who grasps one hand and also clasps the other around the patient's elbow. The subject's upper arm is pushed to and fro and the humerus is externally rotated. The degree of resistance from normal to extreme rigidity is scored as follows.	
**0** = Normal	☐
**1** = Slight stiffness and resistance	☐
**2** = Moderate stiffness and resistance	☐
**3** = Marked rigidity with difficulty in passive movement	☐
**4** = Extreme stiffness and rigidity with almost a frozen shoulder	☐

**4. ELBOW RIGIDITY:**<br>The elbow joints are separately bent at right angles and passively extended and flexed, with the subject's biceps observed and simultaneously palpated. The resistance to this procedure is rated. (The presence of cogwheel rigidity is noted separately.)	
**0** = Normal	☐
**1** = Slight stiffness and resistance	☐
**2** = Moderate stiffness and resistance	☐
**3** = Marked rigidity with difficulty in passive movement	☐
**4** = Extreme stiffness and rigidity with almost a frozen shoulder	☐

SIMPSON-ANGUS SCALE (SAS)

5. WRIST RIGIDITY or Fixation of position:
The wrist is held in one hand and the fingers held by the examiner's other hand, with the wrist n1oved to extension, flexion and ulner and radial deviation:

**0** = Normal	☐
**1** = Slight stiffness and resistance	☐
**2** = Moderate stiffness and resistance	☐
**3** = Marked rigidity with difficulty in passive movement	☐
**4** = Extreme stiffness and rigidity with almost a frozen shoulder	☐

6. LEG PENDULOUSNESS:
The patient sits on a table with his legs hanging down and swinging free. The ankle is grasped by the examiner and raised until the knee is partially extended. It is then allowed to fall. The resistance to falling and the Jack of swinging form the basis for the score on this item:

**0** = The legs swing freely	☐
**1** = Slight diminution in the swing of the legs	☐
**2** = Moderate resistance to swing	☐
**3** = Marked resistance and damping of swing	☐
**4** = Complete absence of swing	☐

SIMPSON-ANGUS SCALE (SAS)

**7. HEAD DROPPING:** The patient lies on a well-padded examining table and his head is raised by the examiner's hand. The hand is then withdrawn and the head allowed to drop. In the normal subject the head will fall upon the table. The movement is delayed in extrapyramidal system disorder and in extreme parkinsonism it is absent. The neck muscles are rigid and the head docs not reach the examining table. Scoring is as follows:	
**0** = The head falls completely with a good thump as it hits the table	☐
**1** = Slight slowing in fall, mainly noted by lack of slap as head meets the table	☐
**2** = Moderate slowing in the fall quite noticeable to the eye	☐
**3** = Head falls stiffly and slowly	☐
**4** = Head does not reach the examining table	☐

**8. GLABELLA TAP:** Subject is told to open eyes wide and not to blink. The glabella region is tapped at a steady, rapid speed. The number of times patient blinks in succession is noted:	
**0** = 0 - 5 blinks	☐
**1** = 6 – 10 blinks	☐
**2** = 11 – 15 blinks	☐
**3** = 16 - 20 blinks	☐
**4** = 21 and more blinks	☐

SIMPSON-ANGUS SCALE (SAS)

**9. TREMOR**: Patient is observed walking into examining room and is then reexamined for this item:	
**0** = Normal	☐
**1** = Mild finger tremor, obvious to sight and touch	☐
**2** = Tremor of hand or arm occurring spasmodically	☐
**3** = Persistent tremor of one or more limbs	☐
**4** = Whole body tremor	☐

**10. SALIVATION:** Patient is observed while talking and then asked to open his mouth and elevate his tongue. The following ratings are given:	
**0** = Normal	☐
**1** = Excess salivation to the extent that pooling takes place if the mouth is open and the tongue raised.	☐
**2** = When excess salivation is present and might occasionally result in difficulty in speaking	☐
**3** = Speaking with difficulty because of excess salivation	☐
**4** = Frank drooling	☐
